# Retraction: Effectiveness of some novel heterocyclic compounds as corrosion inhibitors for carbon steel in 1 M HCl using practical and theoretical methods

**DOI:** 10.1039/d5ra90076j

**Published:** 2025-06-12

**Authors:** Abd El-Aziz S. Fouda, Samir A. Abd el-Maksoud, Elsherbiny H. El-Sayed, Hazem A. Elbaz, Ashraf S. Abousalem

**Affiliations:** a Chemistry Department, Faculty of Science, Mansoura University Egypt asfouda@hotmail.com +20 50 2202264 +20 50 2365730; b Chemistry Department, Faculty of Science, Port Said University Egypt; c Egyptian Natural Gas Company (GASCO) Egypt hazem.elbazz@gasco.com.eg

## Abstract

Retraction of ‘Effectiveness of some novel heterocyclic compounds as corrosion inhibitors for carbon steel in 1 M HCl using practical and theoretical methods’ by Abd El-Aziz S. Fouda *et al.*, *RSC Adv.*, 2021, **11**, 19294–19309, DOI: https://doi.org/10.1039/D1RA03083C.

The Royal Society of Chemistry hereby wholly retracts this *RSC Advances* article due to concerns with the reliability of the data.

The AFM images in Fig. 10A and B were the same as those published by the authors in other papers.^1–10^ The authors stated that the AFM imaging was carried out outside the faculty and was distributed across the research group.

The authors alerted us to this error and requested a correction for these images, however given the significance of these concerns, the Editor has lost confidence that the findings presented in this paper are reliable.

The author Ashraf S. Abousalem has also requested their affiliation is updated, as their 2nd affiliation was not where the work was conducted and should not have been included on the manuscript.

This retraction supersedes the information provided in the Expression of Concern related to this article.

The authors were informed about the retraction of the article, Abd El-Aziz S. Fouda disagreed with the retraction on behalf of all authors, but the other authors have not indicated whether they agree with the decision to retract.

Signed: Laura Fisher, Executive Editor, *RSC Advances*

Date: 4th June 2025

**References**

1 A. S. Fouda, M. A. Ismail, A. A. Al-Khamri and A. S. Abousalem, *J. Mol. Liq.*, 2019, **290**, 111178.

2 A. E.-A. S. Fouda, M. A. Ismail, A. A. Al-Khamri and A. S. Abousalem, *Chin. J. Chem. Eng.*, 2021, **40**, 197–217.

3 G. E. Badr, A. Ali and A. S. Fouda, *Int. J. Electrochem. Sci.*, 2021, **16**, 210832.

4 S. Y. Al-Nami and A. E.-A. S. Fouda, *Int. J. Electrochem. Sci.*, 2020, **15**, 535–547.

5 A. E.-A. S. Fouda, S. A. Abd El-Maksoud, E. H. El-Sayed, H. A. Elbaz and A. S. Abousalem, *RSC Adv.*, 2021, **11**, 13497–13512.

6 A. E.-A. S. Fouda, H. H. Al-Zehry and M. Elsayed, *J. Bio- Tribo-Corros.*, 2018, **4**, 23.

7 A. S. Fouda, A. El-Askalany, A. T. El-Habab and S. Ahmed, *J. Bio- Tribo-Corros.*, 2019, **5**, 56.

8 A. S. Fouda, M. N. El-Haddad, M. A. Ismail and A. Abd Elgyed, *J. Bio- Tribo-Corros.*, 2019, **5**, 73.

9 A. S. Fouda, K. Shalabi and M. S. Shaaban, *J. Bio- Tribo-Corros.*, 2019, **5**, 71.

10 A. E.-A. S. Fouda and M. Eissa, *J. Bio- Tribo-Corros.*, 2020, **6**, 104.

